# Platelet-Rich Plasma, Especially When Combined with a TGF-β Inhibitor Promotes Proliferation, Viability and Myogenic Differentiation of Myoblasts *In Vitro*

**DOI:** 10.1371/journal.pone.0117302

**Published:** 2015-02-13

**Authors:** Robi Kelc, Martin Trapecar, Lidija Gradisnik, Marjan Slak Rupnik, Matjaz Vogrin

**Affiliations:** 1 Department of Orthopaedic Surgery, University Medical Center Maribor, Maribor, Slovenia; 2 Institute of Physiology, Faculty of Medicine, University of Maribor, Maribor, Slovenia; Leiden University Medical Center, NETHERLANDS

## Abstract

Regeneration of skeletal muscle after injury is limited by scar formation, slow healing time and a high recurrence rate. A therapy based on platelet-rich plasma (PRP) has become a promising lead for tendon and ligament injuries in recent years, however concerns have been raised that PRP-derived TGF-β could contribute to fibrotic remodelling in skeletal muscle after injury. Due to the lack of scientific grounds for a PRP -based muscle regeneration therapy, we have designed a study using human myogenic progenitors and evaluated the potential of PRP alone and in combination with decorin (a TGF-β inhibitor), to alter myoblast proliferation, metabolic activity, cytokine profile and expression of myogenic regulatory factors (MRFs). Advanced imaging multicolor single-cell analysis enabled us to create a valuable picture on the ratio of quiescent, activated and terminally committed myoblasts in treated versus control cell populations. Finally high-resolution confocal microscopy validated the potential of PRP and decorin to stimulate the formation of polynucleated myotubules. PRP was shown to down-regulate fibrotic cytokines, increase cell viability and proliferation, enhance the expression of MRFs, and contribute to a significant myogenic shift during differentiation. When combined with decorin further synergistc effects were identified. These results suggest that PRP could not only prevent fibrosis but could also stimulate muscle commitment, especially when combined with a TGF-β inhibitor.

## Introduction

Musculoskeletal injuries that result in the necrosis of muscle fibres are frequently encountered in clinical and sports medicine [1,2]. Despite their clinical significance, current therapeutic options remain rather conservative and include the R.I.C.E. (rest, ice, compression, elevation) principle or the controversial therapy using corticosteroids as well as non-steroidal anti-inflammatory drugs [3].

Injured skeletal muscle has regenerative capacities and can repair spontaneously; however, this process is often incomplete because of overgrowth of the extracellular matrix and the deposition of collagen, which leads to significant fibrous scarring [4,5,6]. Fibrotic remodelling further limits the functionality of the muscle and represents a significant risk factor for the injury to recur.

Platelet-rich plasma (PRP), an autologous platelet concentrate, has gained popularity for the therapy of tendon and ligament injuries [7] despite few and limited relevant scientific reports about its equivalent therapeutic efficiency. PRP is isolated by the centrifugation of whole blood, obtained from an individual, allowing extraction of thrombocytes rich in granules containing various growth factors [8]. As such, the patient-derived endogenous preparation is theoretically perfect to be administered locally at the site of the tissue injury. Several individual PRP-derived growth factors have positive regenerative effects in muscle healing [9,10,11,12]; nonetheless, PRP-derived TGF-β could potentially stimulate fibrosis as shown previously [1,13]. Because of its presence in PRP, its application into skeletal muscle raises concerns due to the risk of even greater fibrotic remodelling of the tissue [14].

**Figure 1:**
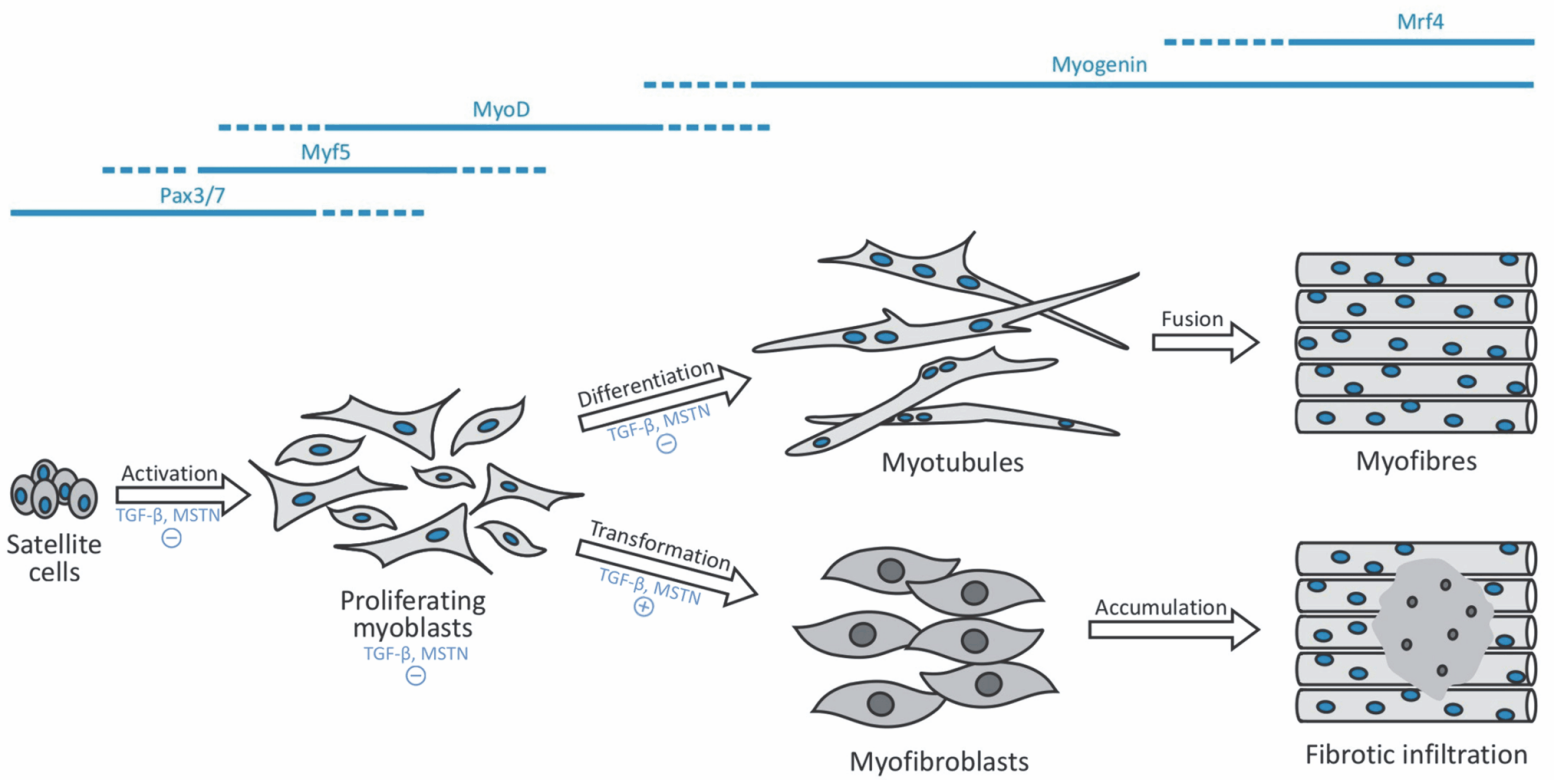
Schematic representation of muscle regeneration on the regulatory level. During skeletal muscle regeneration various MRFs are being expressed (in blue). Satellite cells differentiate into myoblasts which proliferate and either further differentiate into polynucleated myotubules or transform into myofibroblasts. TGF-β and MSTN play an important role in inhibiting/stimulating these steps (marked with +/- symbols).

Fibrotic effects of TGF-β are balanced by decorin, a component of the extracellular matrix of all collagen-containing tissues [15]. It has been shown that decorin inhibits both TGF-β as well as myostatin (MSTN), which is another, skeletal muscle-specific, member of the TGF-β superfamily [16,17,18,19,20]. Both cytokines up-regulate the expression of each other [19] and inhibit the activation of satellite cells [14,21,22], myoblast proliferation [19,23] as well as their myogenic differentiation [13,24,25]. Alternatively they promote fibroblast commitment [1,5,19] (Fig. 1). We hypothesise that by the simultaneous use of the anitfibrotic agent decorin with PRP we might reduce TGF-β-dependent fibrotic scaring while at the same time stimulating muscle regeneration via the introduction of homologous growth factors.

A typical feature during muscle differentiation are transient changes in the expression levels of various muscle specific transcription factors [26] (Fig. 1). Myogenic regulatory factors (MRFs) such as Pax3/7, Myf5, MyoD, myogenin and Mrf4 are expressed exclusively in skeletal muscle [27] and govern the expression of multiple genes during myogenesis [28,29]. MyoD is required for the determination of skeletal myoblasts whereas myogenin acts later in the program, likely as a terminal differentiation factor [27]. Although it has been shown that decorin alters the expression of MRFs in skeletal muscle after injury, such a mode of action has not yet been studied with PRP. Furthermore, despite the results from two recent studies showing positive effects of PRP on skeletal muscle proliferation, no data about TGF-β and MSTN expression has been provided [30,31].

In our study we used a human CD56 positive myoblast cell line and evaluated the potential of PRP alone and in combination with decorin, a TGF-β inhibitor to alter myoblast proliferation, metabolic activity, TGF-β and myostatin activation and expression of myogenic regulatory factors (MRFs). Imaging flow cytometry enabled us to create a valuable and unique insight into the ratio of quiescent, activated and terminally committed single myoblasts in treated versus control cell populations. Finally high-resolution confocal microscopy validated the potential of PRP and decorin to stimulate the formation of desmin-expressing polynucleated myotubes.

## Materials and Methods

### Cell culture isolation and cultivation

We used the human CD56^+^ positive myoblast cell line (hMC) [32,33] previously characterized and stored at the Institute of Physiology, University of Maribor. The hMC cell line was maintained in DMEM (Life Technologies Ltd, Paisley, UK) including 100 Units/ml of penicillin, 1 mg/ml of streptomycin and 2 mmol of L-glutamine, as well as 20% bovine serum (Life Technologies Ltd, Paisley, UK) at 37°C in an atmosphere of 5% CO_2_. The medium was routinely changed every three days to the point of experimentation. Furthermore, during experiments DMEM was used without serum to exclude the influence of contained growth factors and to simulate conditions after injury as previously described by Li et al [30], while PRP and decorin (R&D Systems, Minneapolis, MN, USA) were added to non-control wells.

### Preparation of PRP-derived growth factors

PRP derived from various donors was prepared as previously described by Doucet et al. and Schallmoser et al. [34,35,36]. All participants provided their written consent to participate in the study. The consent procedure and the study were approved by the Slovenian national ethics committee and the institutional review board at the University Medical Centre Maribor. One hundred ml of blood was collected from five single blood donations. Blood was drawn into a tube containing 10 mL Acid Citrate Dextrose (ACD-A) anticoagulant. Five mL of PRP was then prepared using a Magellan Plasma Separator (Medtronic, Minneapolis, USA) according to the manufacturer's protocol. Furthermore, we froze the PRP units to-80°C¸without further manipulation. This was followed by thawing of the units in a water bath at 37°C until ice clots disappeared. We repeated this process 5 times in order to lyse the platelets and release the growth factors. Mechanical lysis was the method of choice in order to preserve chemically unhampered samples. The solution was centrifuged for 10 minutes at 1,500 rpm, and the supernatant was used for ultrafiltration (0.22 μm filter size) in order to remove any residual particles as these tend to aggregate and may induce alloimmunization. One point five mL of suspension containing growth factors was diluted with DMEM (Sigma-Aldrich, Grand Island, USA) in order to make 5%, 10% and 20% solutions of PRP-derived growth factors.

### Viability of treated cell cultures

To determine the effect of PRP-derived growth factors and decorin on the viability of hMC we performed a Tetrazolium [3-(4,5-Dimethylthiazol-2-Yl)-2,5-Diphenyltetrazolium Bromide] (MTT) assay. The cells were seeded at 10,000 cells/well in 96-well plates at 37°C in a humidified CO_2_ incubator until they were confluent. DMEM prepared with different concentrations of PRP exudates (5%, 10%, and 20%) and decorin (10 ng/mL, 25 ng/mL, and 50 ng/mL) were added to the cells, which were further cultured for 4 hours. MTT 5 mg/mL (Sigma) was used for the quantitative determination of cell viability as previously described [37,38].

### Proliferative ability of exposed cell cultures

To perform the cell proliferation assay, hMCs were separately seeded at 10,000 cells/well in 96-well plates at a concentration of 30 viable cells per well. DMEM prepared with different concentrations of PRP exudates (5%, 10%, and 20%) and decorin (10 ng/mL, 25 ng/mL, and 50 ng/mL) were added and incubated for 7 days at 37°C in an atmosphere of 5% CO_2_. After incubation we stained the cells with crystal-violet and measured the absorbance at 595 nm.

### Enzyme-Linked Immunosorbent Assay

Cells were seeded at 10,000 cells/well on 96-well plates. After 48 hours of incubation with 10% and 20% PRP-exudate and decorin (25 μg/mL) the TGF-β and MSTN levels in supernatants were assayed using commercially available enzyme-linked immunosorbent kits (Invitrogen Co., Camarillo, USA and USC Life Science Inc., Wuhan, China) following the manufacturer's protocol. Additionally, we measured the TGF-β concentration in a PRP exudate sample only to show its presence before applying it to the culture. Triplicates were performed for all assays.

### Myogenic differentiation

For myogenic differentiation, myoblasts were seeded in 4-well chamber slides at 10,000 cells/well and 48-well plates at 5,000 cells/well. After 24 hours PRP-exudate, decorin and their combination was added to DMEM without bovine serum, while no drug was added to the control group. After immunostaining, the cells were prepared for microscopical examination and Image Stream single-cell analysis to evaluate the expression of cell surface markers such as CD56 and the myogenic markers MyoD, Myogenin and desmin as further described below.

*Confocal imaging*. Immunocytochemical staining was performed as previously described [39]. Briefly, the cells were seeded in 4-well chamber slides and cultured with a 10% PRP exudate, decorin 25 ng/mL and their combination. After 4 days the cells were fixed, permeabilized and stained with primary and secondary antibodies against desmin (FITC), α-tubulin (goat-anti rabbit IgG Cy3), and nuclei (DRAQ5), all obtained from Abcam (Cambridge, UK). Triplicates were performed for the analysis. Optical images were acquired at the center of chamber slides where the cell density is at its highest using a Leica TCS SP5 II confocal microscope (Leica Microsystems Ltd., Mannheim, Germany) and analyzed using Photoshop CS6 (Adobe, San Jose, USA), where a “Color Range” tool was used in combination with a histogram palette to count the pixels that correspond to desmin-positive areas in an image.

*Imaging multicolor flow cytometry*. In order to quantitatively determine myogenic differentiation of cultured myoblasts, we analyzed the cells using the imaging flow cytometer ImageStreamX (Amnis Corporation, Seattle, USA). After harvesting, followed by permeabilization, we stained the cells simultaneously with antibodies against CD56 (CD56-APC conjugate; BD Pharmingen, Heidelberg, Germany), MyoD (goat MyoD and bovine anti-goat IgG PE conjugate; Santa Cruz GmbH, Heidelberg, Germany) and myogenin (AlexaFluor 488; R&D Systems, Abingdon, UK) according to the manufacturer’s protocols. We acquired cell images of 5,000 events per sample at 40 x magnification using 488 nm and 658 nm lasers and fluorescence was collected using three spectral detection channels. For triple-stained cells (CD56, MyoD and Myogenin), three single-stained controls were used to compensate fluorescence between channel images. Cell images were analyzed with the IDEAS image-analysis software (Amnis). First, we gated the aspect ratio versus cell area to isolate a population of single cells on a bivariate plot. Cells within the focal plane were further selected using a two-dimensional plot of image contrast versus root-mean-squared (rms) gradient. We scouted for the presence of MyoD and myogenin exclusively in CD56 positive cells by measuring the intensity of each probe. Results are expressed as percentage ratios between the cells positive for CD56 only, those additionally positive for MyoD and those expressing also myogenin or myogenin alone in each treated and untreated population of cells, respectively.

### Statistical analysis

Data collected was analyzed using the Statistical Package for Social Sciences (SPSS) version 16. All of the results from this study are expressed as the mean +/- S.D. The differences between means were considered statistically significant if *p* < 0.05. Comparison between groups was made by a Student’s t test, two-way ANOVA for multiple comparisons and Bonferroni post-hoc analysis. A Chi^2^ test was used to analyze the results from image flow cytometry. SPSS software was used for statistical analysis.

## Results

### PRP enhances viability of hMCs

**Figure 2:**
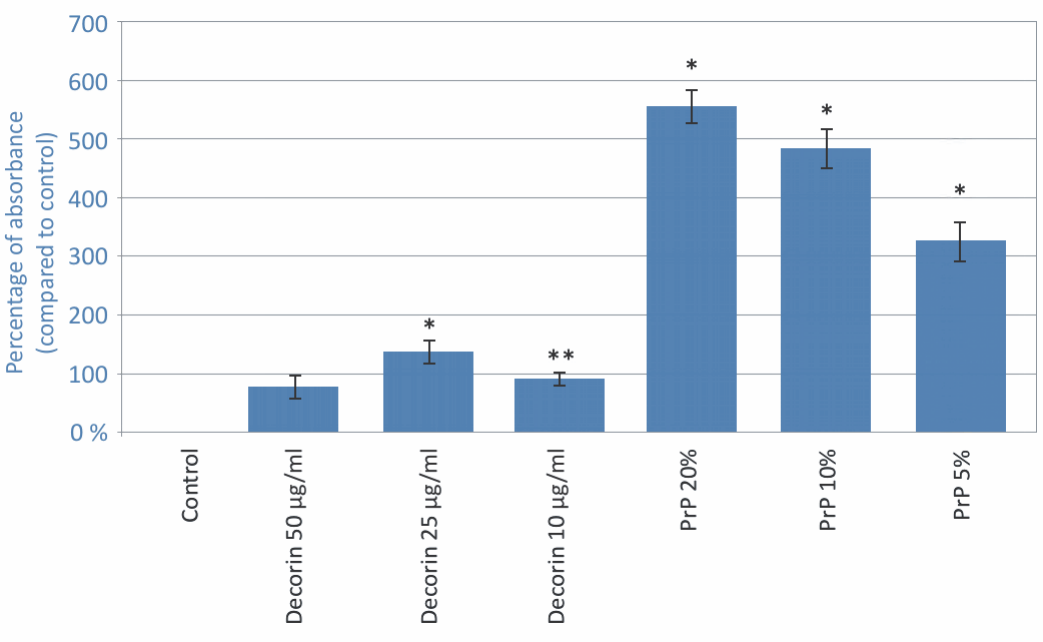
MTT assay. Three independent tests were performed and the results were expressed by the mean ratios (%, ± SD) of absorbance in treated wells to those in control wells. ANOVA, *p<0.001, **p<0.05 compared to control.

The mitochondrial activity of cells, as determined by the MTT assay, was significantly increased (p<0.001) after exposure to tested concentrations of PRP exudates. Similarly, viability was elevated in all tested concentrations of decorin, except 50 ng/ml (Fig. 2). PRP 20% and 10% exudates enhanced the viability of cells to more than 400% when compared to the control (p<0.001). The viability of cells treated with PRP exudates was also significantly higher when compared to both decorin concentrations of 25 ng/ml and 50 ng/ml (p<0.001) whereas there was no significant difference between 10% and 20% PRP exudate concentrations.

### PRP enhances the proliferative ability of hMC

**Figure 3:**
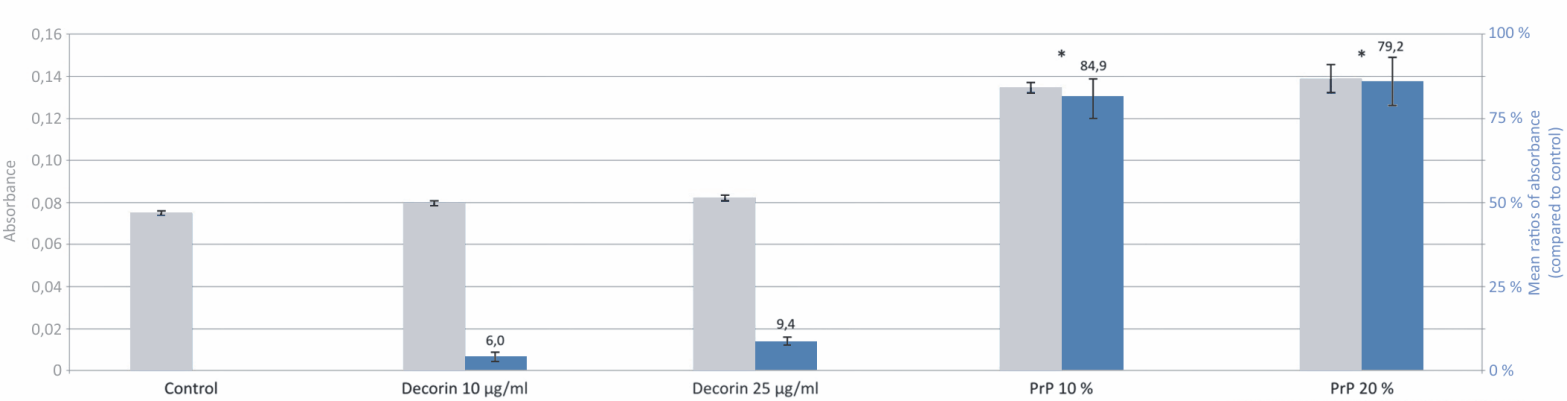
Myoblast proliferation rate. The figure shows the proliferation of myoblasts after 7 days of incubation with DMEM supplemented with decorin 10 ng/ml, decorin 25 ng/ml, 10% PRP exudate and 20% PRP exudate. Three independent tests were performed and the results are expressed by the absolute absorbance values (in blue) and mean ratios (%, ± SD) of absorbance in treated wells to those in control wells (in grey). ANOVA, *p<0.001 compared with control.

We performed a crystal violet assay to determine the proliferative rate of cells incubated for 7 days with two different decorin and PRP exudate concentrations (Fig. 3). Both decorin concentrations did not show a significant effect on cell proliferation compared to the control; however, cultivation with both PRP exudates leads to a 5-fold increase in cell proliferation (p<0.001) with no significant differences between them.

### PRP and decorin—both and in combination down-regulate TGF-β and MSTN expression by hMCs

**Figure 4:**
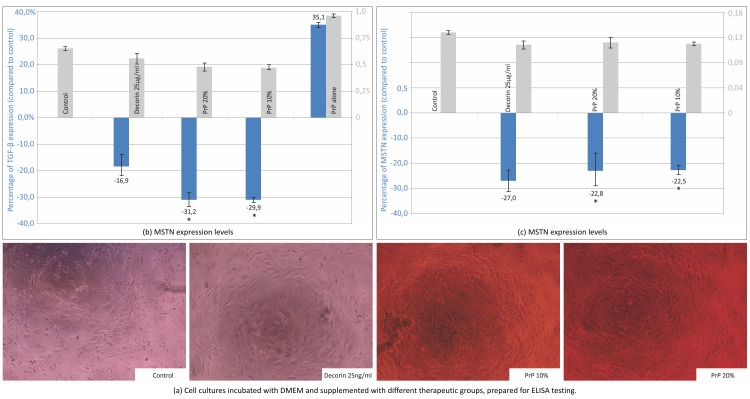
TGF-β and myostatin expression. (a) Cultured myoblasts incubated with DMEM and supplemented with decorin 10 ng/ml, decorin 25 ng/ml, 10% PRP exudate and 20% PRP exudate (ELISA). Cytokine expression was measured after 48 hours of incubation. (b): TGF-β expression. (c): MSTN expression. Three independent tests were performed and the results were expressed by the absolute absorbance values (in grey) and mean ratios (%, ± SD) of absorbance in treated wells to those in control wells (in blue). ANOVA, p<0.001 compared with control, *p<0.005 compared with decorin. In PRP group, no cells were growing in the well in order to determine the TGF-β content in PRP.

Decorin was shown to down-regulate the expression of TGF-β when compared to the control by more than 15% (0.552±0.014 vs. 0.676±0.008 p<0.001) but significantly less than both 10% and 20% PRP exudate concentrations (0.466±0.0.017 and 0.467±0.00027 p<0.005). Tested PRP exudates significantly down-regulated TGF-β expression by more than 30% (p<0.001) (Fig. 4). The pure PRP exudate sample is more than 30% (0.913±0.011 vs. 0.676±0.008, p<0.001) richer in TGF-β concentration than a control sample of cultivated muscle cells confirming that PRP represents an exogenous source of TGF-β.

Similarly, the MSTN expression levels were significantly down-regulated by both decorin and PRP exudates (Fig. 4). MSTN levels of cells treated with decorin were decreased by 28.4% (p<0.001) and 23.1% by PRP (p<0.001) when compared to the control group. There was no significant difference between both PRP exudate concentrations withregard to MSTN expression.

**Figure 5:**
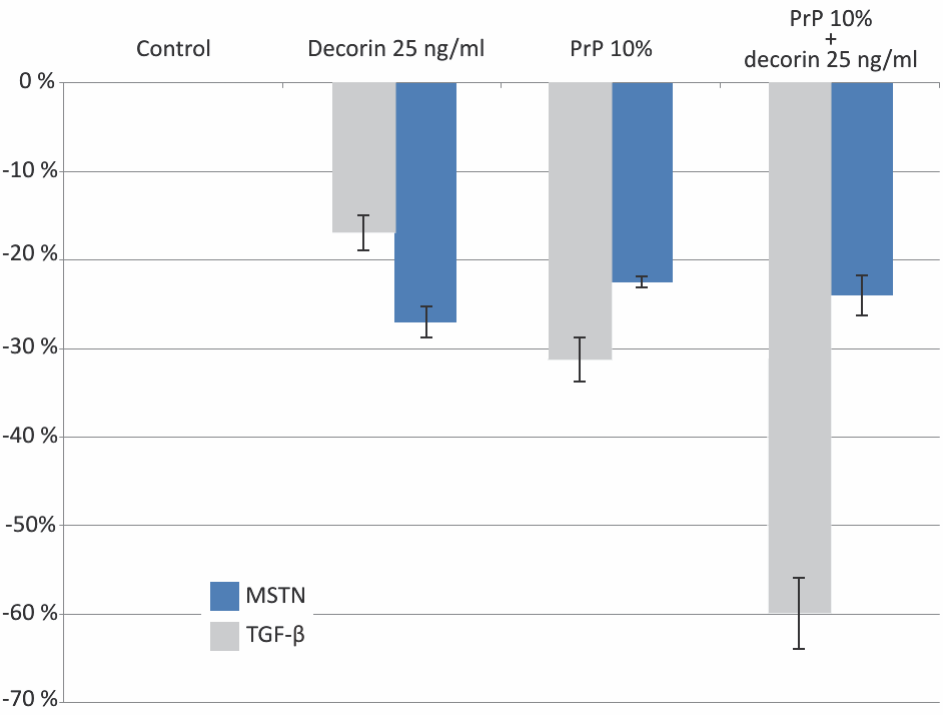
TGF-β and MSTN expression (ELISA). Three independent tests were performed for each cytokine and the results are expressed by the mean ratios (%) of absorbance in treated wells to those in control wells. ANOVA, p<0.001 compared to control.

Since no significant differences in cell viability and cytokine expression of 10% and 20% PRP exudates were found, we performed further experiments with a lower exudate concentration (10%). When cultivating cells with a combination of decorin 25 ng/ml and 10% PRP exudate, the TGF-β cytokine expression was further down-regulated by 59.9% when compared to the control and twice as much when treated with PRP exudate or decorin alone (Fig. 5). MSTN levels were decreased by 24% compared to the control but were not significantly lower than in cells treated with PRP exudate or decorin alone.

### PRP and decorin act synergistically towards myogenic differentiation of hMCs

**Figure 6:**
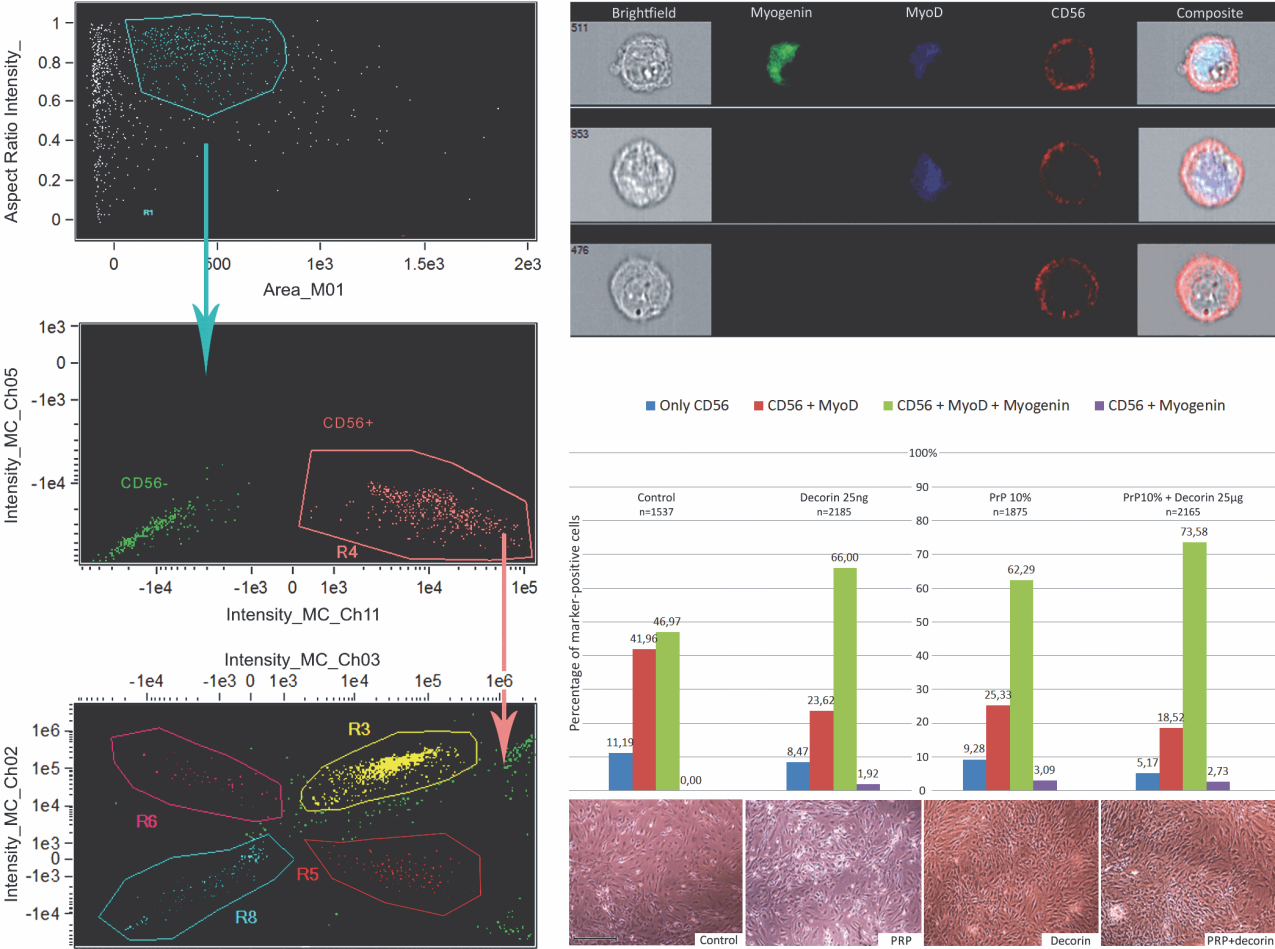
Myogenic differentiation of hMC. Each sample consisted of 5,000 cells that were hierarchically gated according to the expression of specific markers (top right). Top left: Aspect ratio versus cell area gated on a bivariate plot in order to isolate single cells; middle left: subgroup of previously gated cells in focal plane positive with CD56 surface marker selected using a two-dimensional plot of image contrast versus root-mean-squared (rms) gradient; bottom left: presence of MyoD and/or myogenin exclusively in CD56 positive cells by measuring the intensity of each probe. Bottom right: Results are expressed as percentage ratios between cells positive only for CD56, those additionally positive for MyoD and those expressing also myogenin or myogenin alone in each treated as well as each untreated population of cells. Scale bar = 50 μm.

ImageStream analysis revealed the differences in myogenic differentiation between the control, PRP and decorin treated groups, as well as their combinations. We captured and analyzed 5,000 cells of each population positive for CD56. Further cells were hierarchically gated according to co-expression of CD56 and MyoD and/or myogenin as markers of early and late stages of differentiation (Fig. 6).

In the PRP-treated group 39.1% more myogenin positive cells were detected compared to the control. Moreover, there was a 3.09% increase in cells positive only for myogenin, whereas no such cells were found in the control cell population. The population of cells positive only for myogenin is considered as fully differentiated and capable of fusion into myotubes as well as future mucle fibers and is thus of great importance for muscle regeneration. At the same time 20.6% fewer cells remained quiescent (positive only for CD56). Cells positive for both MyoD and myogenin represent the population that shifted significantly towards mature myocites during myogenesis but are not yet fully committed.

While decorin alone led only to a slightly increased differential shift when compared to PRP, it was shown to exert a synergistic effect with PRP. The combination led to a 16.7% increase in cells positive for both myogenin and MyoD (compared to PRP alone). When compared to the control, the combination led to a 60.4% increase in cells positive for myogenin and MyoD and a 46.2% decrease among quiescent cells (only CD56^+^).

**Figure 7:**
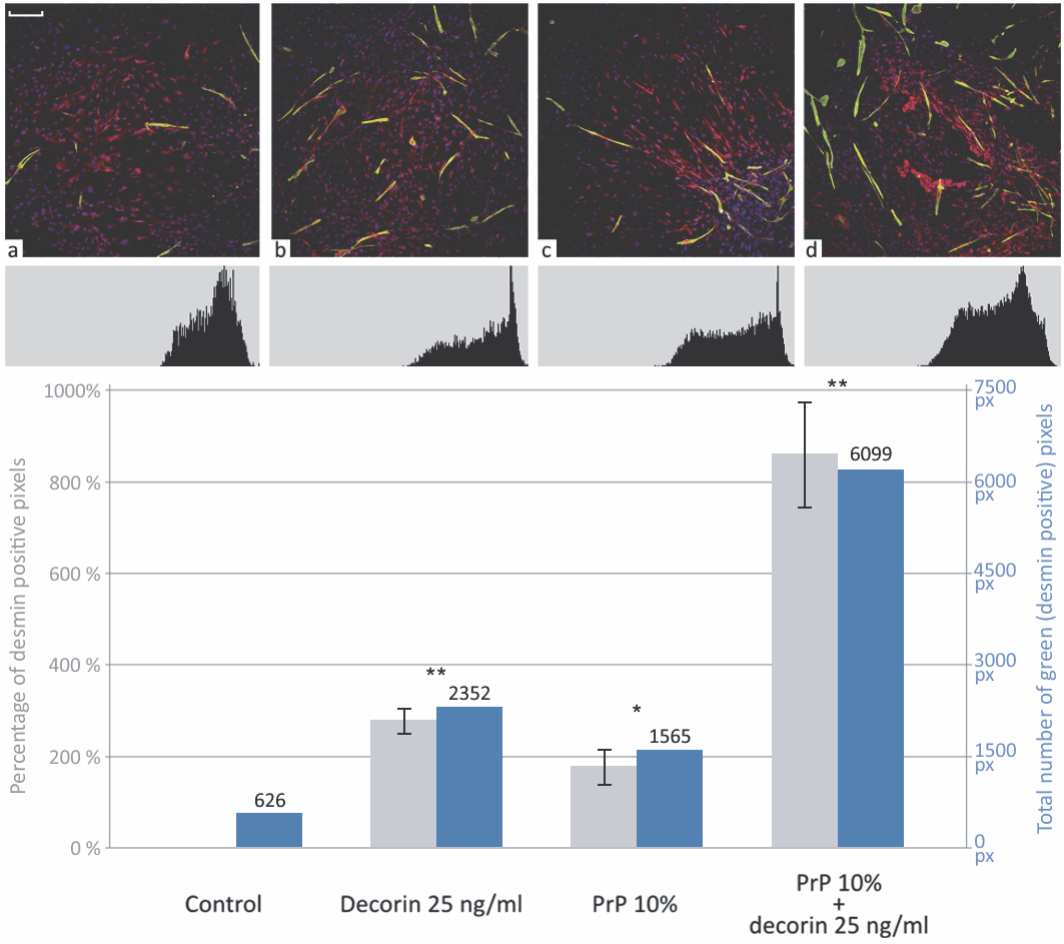
Desmin expressing myotubules. Immunofluorescence staining for merged stained nuclei (blue), α-tubulin (red), and desmin (green). Three independent tests were performed and the results are expressed by the mean values of “Selective color” pixel count (in grey) and mean ratios (%, ± SD) of pixel count in the treated group to those in control groups (in blue). ANOVA, * p<0.01, ** p<0.005 compared with the control. (a) control, (b) decorin-treated group, (c) PRP-treated group, (d) PRP and decorin-treated group. Evident differences can be seen in the polynucleated myotubules count and in desmin expression among the control and PRP and/or decorin treated groups. Scale bar = 200 μm.

In order to visualize the cultured cells and study the presence of desmin-containing myocites we took photomicrographs using a confocal laser microscope. We stained the nuclei, α-tubulin, to visualize the cell cytoplasm, and desmin, an intermediate filament as one of the key markers of myogenic differentiation of myoblasts [39,40]. A color selection tool and histogram analysis were performed using Adobe Photoshop to count the pixels as desmin positive (Fig. 7). A statistically significant up-regulation of desmin expression (p<0.01 for the PRP treated group, p<0.005 for the decorin and PRP + decorin treated groups) was present in all therapeutic groups when compared to the control. While no significant difference was found between the PRP and decorin-treated groups, their combination led to a more than 3-fold increase (p<0.005) of desmin expression when compared to single bioactives.

## Discussion

Despite the limited number of relevant reports confirming its value, platelet-rich plasma (PRP) as a source of autologous growth factors is widely used in therapy of tendinopathies and ligament injuries. Recently, increasing tendencies [41,42] to use PRP to improve skeletal muscle regeneration after injury, raise concerns especially because of one PRPs specific growth factor TGF-β, which is known to impair the process of muscle regeneration. However, our study suggests the opposite. By improving the metabolic activity of myoblasts, we not only excluded the potential cytostatic effect of PRP, but also showed its positive effect on the viability as well as the proliferation of hMC. These findings correlate well with a few previous publications about the positive effects of PRP and various individual growth factors on skeletal muscle regeneration [9,10,11,12,30,43]. The inhibitory effects on TGF-β caused by PRP were also significantly higher when compared to decorin, which was identified as a powerful regulatory agent of muscle regeneration [17,19,20,26].

Although PRP represents a significant source of TGF-β, its overall expression in hMC was down-regulated and was surprisingly greater when compared to hMC treated with decorin, which is one of the most potent TGF-β antagonists [44]. The mechanism behind such an effect remains unknown. It seems as though there is a synergistic connection between multiple PRP-derived growth factors responsible for the outcome and this will be the focus of our future studies.

MSTN is produced rather by skeletal muscle cells and is normally not present in autologous platelet concentrates. Although MSTN does circulate in the blood and may therefore theoretically appear in the preparation no concerns about MSTN-induced fibrotic remodelling after intramuscular PRP injection are found in the literature. However, our data shows that MSTN expression by hMC was down-regulated when treated with PRP in comparison to non-treated cells and almost reached the regulatory level of decorin which is believed to be one of the strongest inhibitors of MSTN activity.

PRP itself down-regulates TGF-β expression in hMC and further reduction is possible with additional inhibition of TGF-β by decorin. After co-cultivation of hMC with PRP and decorin, a 60% decrease in TGF-β expression was identified indicating their synergistic effect.

During muscle regeneration satellite cell progenitors are being activated from the quiescent state followed by the expression of MRFs, while some of the satellite cells remain inactive to provide a further regenerative scaffold. The whole process is mediated by TGF-β and MSTN [45,46] [47,48,49]. Advanced single-cell analysis showed a significant increase of cells expressing MyoD and/or myogenin, which are both MRFs characteristic for myogenic differentiation. Again, synergism of PRP and decorin is evident as the combination of both leads not only to a duplicated count of active satellite cells, but also to a significant shift in myogenic terminal differentiation.

We also showed an evident increase in desmin expression, as well as polynuclear cell count after PRP and decorin treatment. Desmin is a muscle-specific intermediate filament protein expressed early and late in the myogenic program and accumulated during myogenesis *In vitro* [50]. Its expression is directly controlled by MyoD and myogenin and is, as the first expressed cytoskeletal protein during myogenesis, one of the key markers of muscle commitment [51,52]. Our findings correlate with the up-regulation of MyoD and myogenin activation detected using imaging flow cytometry, and serve as additional proof of myogenic shifts towards terminal myoblast differentiation.

## Conclusion

Activation of satellite cells and myogenic differentiation of proliferating myoblasts are two crucial steps for effective skeletal muscle regeneration. Our findings suggest that preparations of autologous growth factors might act as a relevant therapeutic option for skeletal muscle injuries, despite the fact that they represent an additional source of TGF-β. In combination with TGF-β antagonist decorin, the effect of this cytokine can be eliminated. This study presents not only new mechanistic insights into the effects of PRP, but also a possible new therapeutic approach for injured skeletal muscle. Although human studies are due to take place in order to confirm the *In vivo* value of our findings, it seems that decorin-supplemented PRP therapy can be rationalized.
